# Amorphous silicon nanocone array solar cell

**DOI:** 10.1186/1556-276X-7-172

**Published:** 2012-03-06

**Authors:** Subramani Thiyagu, Zingway Pei, Ming-Sian Jhong

**Affiliations:** 1 Graduate Institute of Optoelectronic Engineering, Department of Electrical Engineering, National Chung Hsing University, Taichung, 40227, Taiwan, Republic of China

**Keywords:** Nanocone, amorphous silicon, solar cell

## Abstract

In the hydrogenated amorphous silicon [a-Si:H]-thin film solar cell, large amounts of traps reduce the carrier's lifetime that limit the photovoltaic performance, especially the power conversion efficiency. The nanowire structure is proposed to solve the low efficiency problem. In this work, we propose an amorphous silicon [a-Si]-solar cell with a nanocone array structure were implemented by reactive-ion etching through a polystyrene nanosphere template. The amorphous-Si nanocone exhibits absorption coefficient around 5 × 10^5^/cm which is similar to the planar a-Si:H layer in our study. The nanostructure could provide the efficient carrier collection. Owing to the better carrier collection efficiency, efficiency of a-Si solar cell was increased from 1.43% to 1.77% by adding the nanocone structure which has 24% enhancement. Further passivation of the a-Si:H surface by hydrogen plasma treatment and an additional 10-nm intrinsic-a-Si:H layer, the efficiency could further increase to 2.2%, which is 54% enhanced as compared to the planar solar cell. The input-photon-to-current conversion efficiency spectrum indicates the efficient carrier collection from 300 to 800 nm of incident light.

## Introduction

The solar energy is a renewable energy and is expected to alleviate the progress of global warming. However, the cost to produce electricity by solar energy harvesting is still higher than the traditional method, such as thermal power generation by burning coal and petroleum or the hydro-electrical power generation. Thin-film silicon solar cell is one of the candidates to achieve low cost requirement. However, to achieve low cost, the low-temperature process makes the thin-film silicon generally be in the form of microcrystalline or becomes amorphous. In this structure, large amounts of traps reduce the carrier's lifetime that limit the photovoltaic performance, especially the power conversion efficiency. The nanowire structure is proposed to solve the low efficiency problem [1]. The light harvesting is along the wire and the carrier collection is along the radial direction. The path for photo-carrier collection and light harvesting is perpendicular. Longer wire could ensure that the solar light be harvested thoroughly while maintaining the efficient carrier collection. Among all materials, the hydrogenated amorphous silicon [a-Si:H] nanowire solar cell has been particularly investigated and predicted to have better photovoltaic performance over planar solar cell [2]. Additional to the efficient carrier collection, the nanostructure surface was expected to have light-trapping behavior that could further increase the total amount of solar energy harvesting in a short nanowire [3-5]. These advantages cause the nanowire solar cell to largely improve the efficiency compared to planar solar cell. However, the randomly grown Si nanowires by bottom-up method are hardly to manufacture a good solar cell device that has higher photovoltaic performance on Si nanowire than planar one is rarely reported [6-8]. Moreover, the combination of the efficient carrier collection and light trapping limits the understanding of the electrical advantages of nanowire solar cell itself. In this work, we prepare the a-Si:H nanostructure by reactive-ion etching [9-12] through a closely packed nanosphere template [13]. In previous study, the low aspect ratio nanocone a-Si:H has negligible light-trapping effect [14]. The light illuminated through glass side instead of surface of the a-Si:H nanocone to make the effect of light-trapping negligible To explore the advantage of the nanostructure, four types of a-Si:H were used to fabricate a-Si:H solar cell. The first one is the planar solar cell without nanostructure used as a reference. The second one is an intrinsic-a-Si:H nanocone solar cell. The third is an intrinsic-a-Si:H nanocone solar cell with H_2 _plasma treatment for 10 min prior to N^+^-a-Si:H deposition. The last one is also an intrinsic-a-Si:H nanocone solar cell with additional 10 nm a-Si:H layer after H_2 _plasma treatment.

## Experimental method

**Figure 1:**
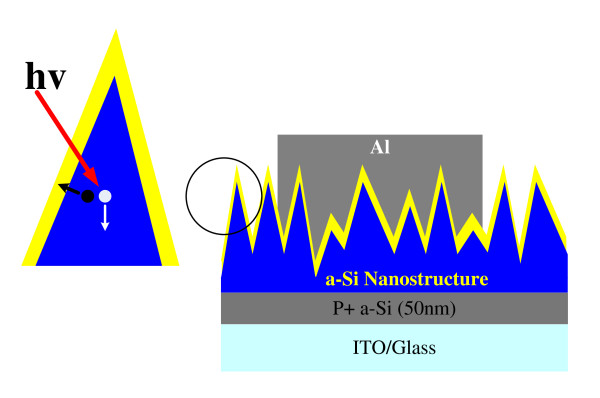
**The schematic structure for a-Si:H-nanocone solar cell**. Enlarged part of the a-Si:H-nanocone structure describes the photogeneration and transport mechanism.

**Figure 2:**
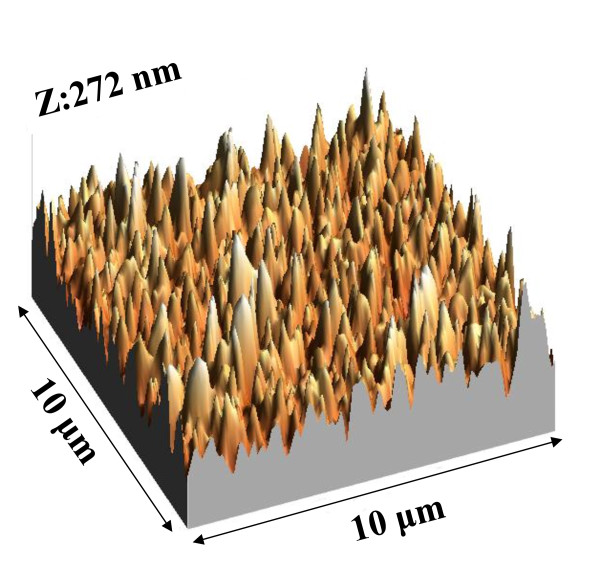
**The AFM surface morphology of a-Si:H nanocone**. The dimension is 10 × 10 μm^2^.

Figure 1 depicts the schematic structure of the proposed a-Si:H random nanocone solar cell. The process flow to fabricate the a-Si:H random nanocone solar cell is described as follows. In the first step, the P^+ ^and instrinsic a-Si:H, 50- and 200-nm thick, respectively, were grown on a cleaned indium-tin oxide/glass substrate by plasma-enhanced chemical vapor deposition [PECVD] at 130°C. A template containing nanospheres was prepared on top of the a-Si:H by nanoscopic phase separation of poly(styrene-*block*-methyl methacrylate) [PS-*b*-PMMA] diblock copolymer. The PS-*b*-PMMA powder was dissolved in toluene at a concentration of 20 mg/ml. After stirring overnight, the PS-*b*-PMMA solution was spin-coated on the a-Si:H and was annealed at 180°C for 24 hours to initiate nanophase separation. The PS-*b*-PMMA films were then immersed in a heated acetic acid at 80°C for 20 min to form the polystyrene [PS] nanospheres. After PS nanosphere formation, the sample was thoroughly rinsed with deionized water and dried in an nitrogen-filled environment. The diameter of the nanospheres is approximately 30 to 50 nm. This template pattern was transferred to the a-Si:H by reactive ion etching [RIE]. The etching power was 250 W with CF_4 _and O_2 _as the etching gasses with a ratio of 20:1. The ratio of etching rate for a-Si:H and PS in CF_4_/O_2 _plasma is approximately 4.5:1. As a result, by carefully controlling the PS thickness (approximately 30 nm), the PS could be completely removed leaving a-Si:H nanostructure. The atomic force microscopy surface morphology of the obtained a-Si:H nanostructure is shown in Figure 2. A random cone-shaped nanotexture was obtained. The reason of the randomly distributed cone-shaped nanostructure is believed due to the lateral chemical etching of a-Si:H under the PS nanospheres and the gradual shrinkage of the size of the PS nanospheres themselves. The height of the random nanocone is approximately 150 nm. With the roughly 30- to 50-nm diameter of the cone tip, the aspect ratio is approximately 3 to 5. The bottom of each a-Si:H-random nanocone is still connected by the P^+ ^a-Si:H layer. After a-Si:H-random nanocone formation, the 45-nm thick N^+ ^a-Si:H was grown in a PECVD chamber at 130°C. The H_2 _plasma treatment was carried at a radio-frequency power of 20 W for 10 min to remove native oxide and passivate the a-Si:H surface. Finally, an Al layer of 100-nm thick was deposited by thermal evaporation as the cathode of the solar cell. Enlarged part of the a-Si:H nanocone structure is also schematically shown in the Figure 1 to describe the photogeneration and transport mechanism. While the light was illuminated to the a-Si:H layer, the photon was absorbed and generated electron-hole pair. The hole moved toward the bottom P^+ ^a-Si:H as in the planar cell. However, the electron moved to the N^+^-a-Si:H layer, nearly perpendicular to the hole movement direction. The electron moving in a short distance than planar cell might cause an efficient collection efficiency for electron that enhances the photocurrent.

## Results and discussion

**Figure 3:**
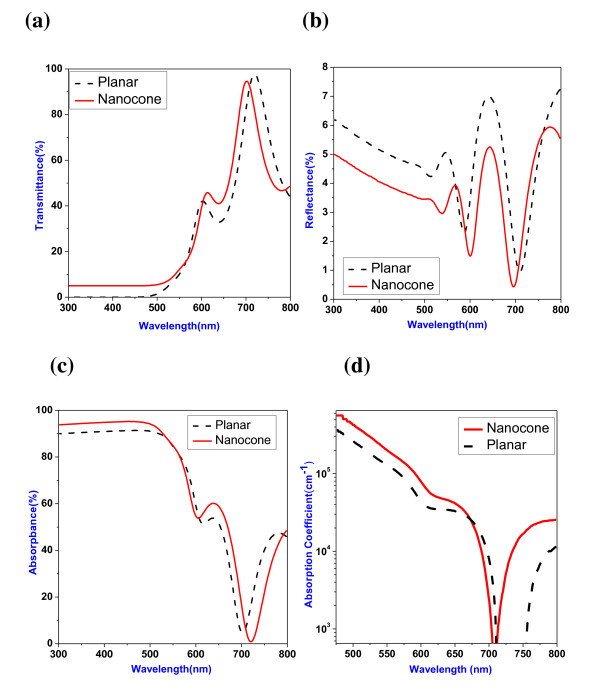
**Transmittance (a), reflectance (b), absorbance (c) and absorption coefficient (d)**. Percentage values are for planar (black broken line) and nanocone (red line) a-Si:H layers.

An ultraviolet-visible-near infrared spectrometer was used to explore the optical behavior of a-Si:H nanocone. Figure 3a depicts the transmittance of a-Si:H planar and nanocone layer. At the wavelength less than 500 nm, the low transmittance [T_0_] indicates the high absorption of a-Si:H. The surface reflectance will affect the amount of light absorption; therefore, the reflectance [R] spectrum was measured and depicted in Figure 3b. For both planar and nanocone a-Si:H, they exhibit similar transmittance and reflectance. Consequently, the absorbance [A] (%), which is calculated through *A *= 1-*R*-*T*_0 _and was depicted in Figure 3c, has similar behavior for planar and nanocone structure. There is no light-trapping behavior in the a-Si:H random nanocone with low aspect ratio [14]. By taking the reflectance [R] and transmittance [T_0_] spectra into account, the absorption coefficient can be calculated by the following equation:

(1) $$T=\frac{T_{0}}{\left(1-R\right)}=e^{-\alpha \times d}$$

, in which T is the absolute transmission, *α *is the absorption coefficient and *d *is the thickness of the intrinsic-a-Si:H layer. Figure 3d depicts the absorption coefficient of planar and nanocone a-Si:H layer. The absorption coefficient for the a-Si:H nanocone is approximately 5 × 10^5^/cm at 500 nm which is slightly higher than the planar structure. The effect of the difference in total amount of light harvesting between planar and nanocone solar cell while exploring the carrier collection efficiency can be minimized by light illumination through the glass side.

**Figure 4:**
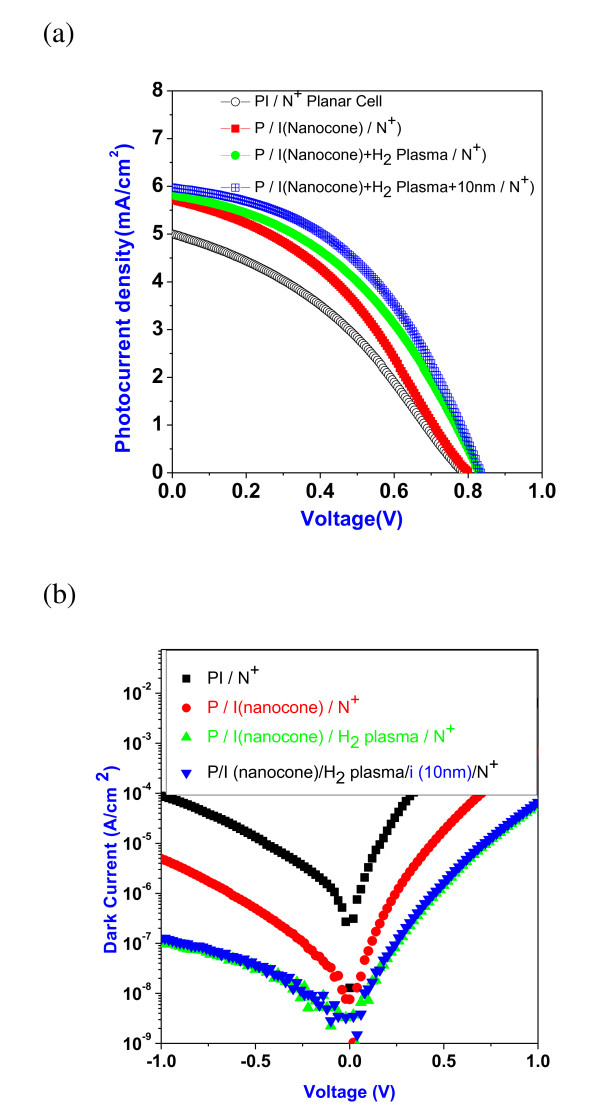
**The current density-voltage characteristics**. Solar cells with 200-nm thick planar-a-Si:H film, 200-nm thick a-Si:H nanocone, 200-nm thick a-Si:H nanocone with 10-min H_2 _plasma treatment, and 200-nm thick a-Si:H nanocone and additional 10-nm a-SiH layer after 10 min H_2 _plasma treatment. (**a**) Photovoltaic properties under AM 1.5 G light illumination (PI/N^+ ^planar cell, circle with center dot; P/I nanocone/N^+^, red square; P/I nanocone + H_2 _plasma/N^+^, green circle; P/I nanocone + H_2 _plasma + 10 nm/N^+^, blue square) and (**b**) under dark condition (PI/N^+^, black square; P/I nanocone/N^+^, red circle; P/I nanocone/H_2 _plasma/N^+^, green triangle; P/I nanocone/H_2 _plasma/i (10 nm)/N^+^, blue inverted triangle).

**Figure 5:**
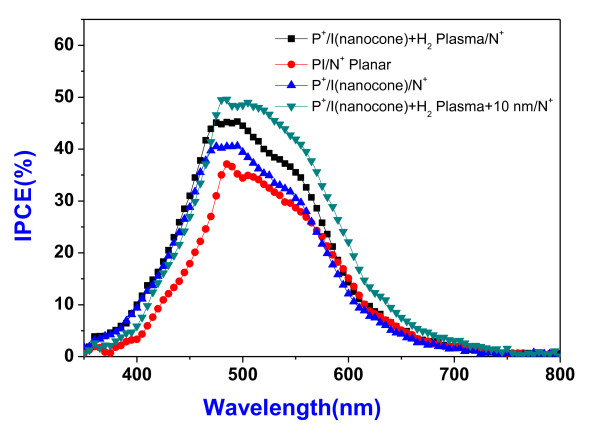
**The input photon-to-current conversion efficiency [IPCE] spectrum current density-voltage characteristics**. IPCE spectrum current density-voltage characteristics of solar cells with 200-nm thick planar-a-Si:H film, 200-nm thick a-Si:H nanocone, 200-nm thick a-Si:H nanocone with 10-min H_2 _plasma treatment, and 200-nm thick a-Si:H nanocone and additional 10-nm a-SiH layer after 10-min H_2 _plasma treatment. P^+^/I nanocone + H_2 _plasma/N^+^, black square; PI/N^+ ^planar, red circle; P^+^/I nanocone/N^+^, blue triangle; P^+^/I nanocone + H_2 _plasma + 10 nm/N^+^, green inverted triangle.

The photovoltaic properties of a-Si:H solar cell was measured by solar simulator under air mass 1.5 G condition. Figure 4a shows the photocurrent density-voltage behavior. The planar pin a-Si:H solar cell exhibit short-circuit current density [Jsc] of 5.0 mA/cm^2 ^and power conversion efficiency [PCE] of 1.43%. The detailed photovoltaic properties were listed in Table 1. With surface nanocone structure, the Jsc increases to 5.7 mA/cm^2 ^which is 14% enhanced. Additionally, the PCE also increases to 1.77% which is 24% enhanced. The short transport path for the electron in the a-Si:H nanocone contributes the additional 0.7 mA/cm^2 ^photocurrent [2]. However, the native oxide and defects on the a-Si:H nanocone after RIE etching may either restrict part of photocurrent transport or act as recombination centers that increase the series resistance as high as 160 Ω·cm^2^. The H_2 _plasma was used to remove the native oxide and passivate the defects. After treating the a-Si:H nanocone surface by H_2 _plasma, the native oxide was removed and the surface defects were passivated [15,16]. This could be understood by the dark current as shown in Figure 4b. The reverse leakage current density was largely reduced after plasma treatment. The series resistance also reduces to around 60 Ω·cm^2^. Additionally, the Jsc further increases to 5.8 mA/cm^2^. The PCE is 2.0%, approximately 40% enhanced over planar solar cell. Another way to reduce the interface state defect density is to cover the surface by a layer of intrinsic a-Si:H. The 10-nm intrinsic a-Si:H was grown after H_2_-plasma treatment. With this additional layer, Jsc further increases to 5.9 mA/cm^2^. The PCE increases to 2.2%, approximately 54% enhanced over planar solar cell. Further analysis of the nanocone solar cell by input photon-to-electron conversion efficiency [IPCE] spectrum could investigate the photoresponse at each wavelength. Figure 5 shows the IPCE results. At short wavelength, the surface was passivated. The higher electrical field in the nanocone structure [17] accelerates the carrier transport that reduces recombination. As a consequence, there is an increase in the IPCE. At long wavelength, the better carrier collection efficiency of the nanocone structure ensures the higher IPCE as compared to the recent publication on the a-Si PIN solar cell with nanodome surface which exhibits efficient light management [18], achieving extensive solar energy conversion efficiency. This work mainly focuses on the transport part. Without light-trapping efficient in nanocone structure, the photocurrent enhancement supports the assumption of efficient carrier collection by the nearly perpendicular light absorption and carrier transportation in nanostructure.

**Table 1:** The detailed photovoltaic properties of a-Si:H nanocone solar cell

Type	PCE (%)	Jsc (mA/cm^2^)/Rsh (Ω·cm^2^)	Fill factor (%)	Voc (V)/Rs (Ω·cm^2^)
PI/N	1.43	5.0/624	36.4	0.78/176
P/I(nanocone)/N	1.77	5.7/572	38.5	0.80/160
P/I(nanocone)/N (H_2 _Plasma)	2.0	5.8/800	41.4	0.83/62
P/I(nanocone)/N (H_2 _Plasma+10 nm i)	2.2	5.9/847	44.3	0.83/61

PCE, power conversion efficiency; Jsc, short-circuit current density; Rsh, shunt resistance; Voc, open-circuit voltage; Rs, series resistance.

## Conclusions

In this work, we propose an amorphous silicon [a-Si] solar cell with a nanocone-array structure which were implemented by PS nanospheres as template for RIE dry etching. The amorphous Si nanocone exhibits 5 × 10^5^/cm absorption coefficient which is similar to the planar a-Si:H layer in our study. The proposed nanocone solar cell could have better carrier collection efficiency and implies an efficiency of 1.77% for a-Si nanocone solar cell which has 24% enhancement over planar solar cell (1.43%). With hydrogen plasma treatment and additional 10-nm a-SI:H layer, the efficiency further increased to 2.2%, which is 54% enhanced as compared to the planar solar cell. This indicates that the a-Si nanostructure could efficiently enhance the photocurrent of the thin-film solar cell.

## Abbreviations

a-Si: amorphous silicon; IPCE: input photon-to-electron conversion efficiency; Jsc: short-circuit current density; PCE: power conversion efficiency; PECVD: plasma enhanced chemical vapor deposition; PS: polystyrene; RIE: reactive ion etching; SEM: scanning electron microscope.

## Competing interests

The authors declare that they have no competing interests.

## Authors' contributions

ZP conceived of the study, participated in its design and coordination, and revised the manuscript. ST drafted the manuscript and carried out the experiments on fabrication of PS nanosphere and optical characterization. MSJ carried out the PECVD experiments. All authors read and approved the final manuscript.
